# Bayesian Analysis of the Glacial‐Interglacial Methane Increase Constrained by Stable Isotopes and Earth System Modeling

**DOI:** 10.1002/2018GL077382

**Published:** 2018-04-22

**Authors:** Peter O. Hopcroft, Paul J. Valdes, Jed O. Kaplan

**Affiliations:** ^1^ Bristol Research Initiative for the Dynamic Global Environment, School of Geographical Sciences University of Bristol Bristol UK; ^2^ Cabot Institute University of Bristol Bristol UK; ^3^ Now at the School of Geography, Earth and Environmental Sciences University of Birmingham Edgbaston UK; ^4^ Max Planck Institute for the Science of Human History Jena Germany; ^5^ ARVE Research SARL Pully Switzerland

**Keywords:** wetlands, Last Glacial Maximum, methane, 13CH4, greenhouse gas, isotopic discrimination

## Abstract

The observed rise in atmospheric methane (CH_4_) from 375 ppbv during the Last Glacial Maximum (LGM: 21,000 years ago) to 680 ppbv during the late preindustrial era is not well understood. Atmospheric chemistry considerations implicate an increase in CH_4_ sources, but process‐based estimates fail to reproduce the required amplitude. CH_4_ stable isotopes provide complementary information that can help constrain the underlying causes of the increase. We combine Earth System model simulations of the late preindustrial and LGM CH_4_ cycles, including process‐based estimates of the isotopic discrimination of vegetation, in a box model of atmospheric CH_4_ and its isotopes. Using a Bayesian approach, we show how model‐based constraints and ice core observations may be combined in a consistent probabilistic framework. The resultant posterior distributions point to a strong reduction in wetland and other biogenic CH_4_ emissions during the LGM, with a modest increase in the geological source, or potentially natural or anthropogenic fires, accounting for the observed enrichment of *δ* ^13^CH_4_.

## Key Points

- For the first time we combine process‐based models of the CH_4_ cycle as priors, with ice core observations in a Bayesian framework
- For the glacial we infer unchanged or increased geological sources, but a halved wetland flux, which is not captured by process modeling
- This suggests missing processes in methane models, with potentially important consequences for confidence in future methane projections

## 1 Introduction

Atmospheric methane (CH_4_) is an important greenhouse gas. Its concentration has risen sharply over the past two centuries, reaching 1,799 ppbv by Common Era (CE) 2010 (Kirschke et al., 2013). This is estimated to have contributed around 25% of the anthropogenic greenhouse gas effect since CE 1750 (Myhre et al., 2013). CH_4_ is also reactive with a lifetime of about 9 years (Prather et al., 2012), and so its abundance indirectly affects the concentrations of other trace gases such as nitrous oxide and ozone.

Observed variations in the growth rate over the past decades are not well understood (e.g., Bousquet et al., 2011; Kirschke et al., 2013). Different studies have implicated changes in biogenic emissions (e.g., Nisbet et al., 2016; Schaefer et al., 2016), lifetime (Rigby et al., 2017) or biomass burning emissions (Worden et al., 2018) in the recent growth rate. Prior to the observational era, changes in atmospheric methane can be accurately reconstructed from gas bubbles recovered in ice cores. During the late Quaternary, CH_4_ mixing ratios are correlated with climate (e.g., Loulergue et al., 2008). Explaining the magnitude of these variations remains a challenge (Hopcroft et al., 2017; Levine, Wolff, Jones, Sime, Valdes, et al., 2011; Murray et al., 2014), reflecting incomplete understanding of the source and sink processes.

The increase in atmospheric CH_4_ concentration from the LGM to the beginning of the Industrial Revolution (hereafter the late preindustrial) is among the largest such changes, with an increase from 375 ppbv to 680 ppbv (Loulergue et al., 2008; Mitchell et al., 2013; WAIS Divide Project Members, 2015). This concentration increase is likely the result of a near doubling of the source of methane (Levine, Wolff, Jones, Sime, Valdes, et al., 2011; Murray et al., 2014). Considerable debate remains over how this occurred (Kaplan et al., 2006; Levine, Wolff, Jones, Sime, Valdes, et al., 2011; Murray et al., 2014; Valdes et al., 2005), because of uncertainties in the response of methane sources and sinks to climate, and in terms of the relative importance of different sources in the late preindustrial era (Hopcroft et al., 2017).

Concurrent changes in the stable isotopes of methane can provide additional constraints, because different sources and sinks of methane have distinct isotopic signatures and fractionation rates (e.g., Bock et al., 2017). Between the LGM and preindustrial, *δ* ^13^CH_4_ became depleted from −43 ± 0.3‰ to −48 ± 0.3‰, with a similar depletion of deuterium of CH_4_ (*δ*DCH_4_) from −79 ± 4‰ to −98 ± 3‰ (Fischer et al., 2008; Möller et al., 2013; Sowers, 2006, 2010).

Fischer et al. (2008) used a box model of atmospheric CH_4_ and CH_4_ isotopes and adjusted the relative contributions of sources and sinks to explain the observed glacial‐interglacial changes. They inferred an increase in wildfire CH_4_ emissions (+18%) at the LGM and a reduction in wetland emissions (−41%) and CH_4_ lifetime (−32%). The latter is in conflict with most model‐based estimates.

More recently, Möller et al. (2013) extended ice core records to 160,000 years Before Present and found that the *δ* ^13^CH_4_ is positively correlated with the atmospheric concentration of CO_2_ and not CH_4_. This suggests that the observed variations of *δ* ^13^CH_4_ likely arise through the effect of CO_2_ and climatic changes on the isotopic fractionation rates of CH_4_ sources or sinks (Whiticar & Schaefer, 2007), rather than only by changes in the relative importance of the different source types, as previously assumed (Fischer et al., 2008). Möller et al. (2013) suggest that shifts in the isotopic signature of tropical wetland CH_4_ are the main contributor.

Here we use process‐based Earth System model simulations of the late preindustrial and LGM methane cycles (Hopcroft et al., 2017, H17 hereafter), to better understand the change in CH_4_. We calculate the implied isotopic composition of methane, including the environmentally driven change in the isotopic discrimination of vegetation and hence the isotopic signature of wetland and biomass burning emissions. We combine these results in a Bayesian framework, which allows us to incorporate uncertainty estimates and include prior information.

## 2 Incorporating Process‐Based Estimates of Changes in Methane Sources and Lifetime Into a Model of Atmospheric Methane Stable Isotopes

We use the CH_4_ sources as incorporated in the Earth System model simulations of the late preindustrial and LGM with HadGEM2‐ES (Collins et al., 2011; HadGEM2 Development Team, 2011, and see supporting information), configured with glacial boundary conditions as described previously (Hopcroft & Valdes, 2015; Singarayer & Valdes, 2010; H17). HadGEM2‐ES is a widely used, coupled Earth System model (e.g., Booth et al., 2012; Caesar et al., 2013; Hopcroft & Valdes, 2015; Jones et al., 2011; Kandlbauer et al., 2013). It includes wetlands (Gedney et al., 2004; Marthews et al., 2015) and tropospheric chemistry (O'Connor et al., 2014). Separate models of peatlands (Wania et al., 2010), biomass burning (Kaplan et al., 2016; Pfeiffer et al., 2013), oceans, and termites (Kaplan et al., 2003; Sanderson, 1996) were also used, see the supporting information.

Using the standard wetland scheme, the high‐latitude emissions are likely underestimated, as shown by a comparison of CH_4_ concentrations for the present day (Hayman et al., 2014). Including the peatland flux leads to a stronger high‐latitude source, as well as a larger reduction in total wetland/peatland emissions at the LGM of 42% versus 30% without.

H17 presented three alternative scenarios of LGM fire emissions: standard‐fire (simulated with a process‐based dynamic vegetation model), standard+LGM humans, which additionally incorporates an empirically based estimate of hunter‐gatherer fire activities during the LGM (Kaplan et al., 2016), and low fire, in which LGM fire emissions are arbitrarily set to 10% of late preindustrial values. Here preindustrial fire emissions are scaled based on ice core evidence and other modeling studies (Ferretti et al., 2005; Thonicke et al., 2005), as described in the supporting information.

We introduce a geological source which comprises mud volcanoes, marine seeps, microseepage, and geothermal methane, which are commonly missing from methane inventories (e.g., Etiope et al., 2008). We reduce the ocean source to 1 TgCH_4_/year in light of recent observations (Kirschke et al., 2013) and set both the hydrate and geological terms to 10 TgCH_4_/year (Petrenko et al., 2017; H17). We incorporate OH, soil, and stratospheric sink terms as modeled by H17. We also here include a boundary layer atomic chlorine (Cl) sink, which we set to 3% of the total CH_4_ sink (Allan et al., 2007; Platt et al., 2004). The assumed isotopic signature of each source and the fractionation factors for the four methane sinks are listed in Table 1, along with estimated prior and posterior values.

**Table 1:** Preindustrial and Prior and Posterior LGM Methane Sources and Lifetime

		LGM					
		Prior			Posterior		
Sources (TgCH_4_/year)	Late Preindustrial	Mean	±1 s.d.	ΔLGM (%)	Mean	±1 s.d.	ΔLGM (%)
N extratropical wetland	55	18	4.7	−67	12.0	4.0	−78
Tropical wetland	73	55	14.3	−25	32.0	4.7	−56
S extratropical wetland^a^	11	6.2	—	−44	—	—	
Biomass burning	21	13.5	4.8	−36	16.6	2.0	−21
Termites	20	12	3.1	−40	10.8	3.0	−46
Hydrates	10	10	3.6	0	6.9	2.2	−31
Other geological	10	10	4.6	0	10.5	2.5	5
Oceans^a^	1	0.8	−	−20	−	−	−
Sum	201	126	34	−38	96	18	−52
Total lifetime (yr)	9.7	10.3^b^	0.2	6	10.4^c^	0.1	7

Note. The LGM means and standard deviations are derived from the PDFs shown in the blue curves in Figure 3. The individual prior sink terms are given in Table S4 in the supporting information. Refer to Tables S2 and S3 for isotopic signatures of individual sources and sinks.

a Indicates sources not varied as part of the Bayesian algorithm.

b Calculated using the prior mean LGM fire emissions.

c Calculated using the posterior mean LGM fire emissions. LGM = Last Glacial Maximum.

**Figure 1:**
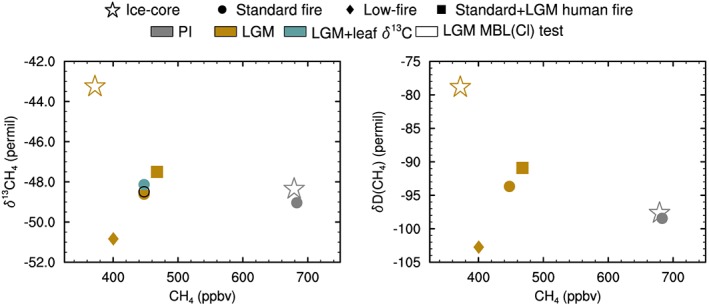
Box model calculations of late preindustrial and Last Glacial Maximum (LGM) CH_4_, *δ* ^13^CH_4_, and *δ* ^13^D(CH_4_). The model is driven with emissions and lifetime derived from HadGEM2‐ES climate‐chemistry simulations. Ice core data (Fischer et al., 2008; Möller et al., 2013; Sowers, 2006, 2010) are shown by star symbols. Three different model fire estimates for the LGM are shown (low‐fire, standard‐fire, and standard with LGM human fire).

We combine these estimates using a three‐box model of atmospheric CH_4_ and stable isotopes as described in the supporting information (Lassey et al., 2000; Miller, 2005). The results show a preindustrial CH_4_ concentration, *δ* ^13^C and *δ*D of 683 ppb, −49.0‰ and −98.4‰ respectively. These are in agreement with ice core measurements, as compared in Figure 1. Using LGM fluxes (with standard‐fire) results in LGM CH_4_, *δ* ^13^C and *δ*D(CH_4_) values of 447 ppbv, −48.6‰, and −93.7‰, respectively, also shown in Figure 1. For both time periods, the box model values are extracted at either the northern or southern box depending on the relevant ice core location, as listed in Table S1. This LGM simulation underestimates both the reduced LGM CH_4_ concentration and shift in isotopes. The low‐fire scenario in which all fire emissions are set to 10% of the late preindustrial results in a better prediction of the LGM CH_4_ change, but at the expense of the *δ* ^13^CH_4_ and *δ*D(CH_4_), which both shift to more isotopically depleted values. The standard‐fire+LGM humans has the opposite effect but is closer to the standard‐fire simulation. As a result, to simplify the presentation we only use the standard‐fire scenario (filled circles in Figure 1) in the following.

We evaluated the potential influence of changes in the Cl sink following the approach of Levine, Wolff, Jones, and Sime (2011). Using monthly fields from late preindustrial and LGM simulations with HadGEM2‐ES (Hopcroft & Valdes, 2015; H17), this results in a small enrichment of *δ* ^13^C at the LGM of 0.12‰, shown in Figure 1.

## 3 Changes in the Isotopic Discrimination by Vegetation and Potential Influence on CH_4_ Emissions

During the LGM the *δ* ^13^C of atmospheric CO_2_ was enriched by 0.1‰ (Schmitt et al., 2012), and thus, this has relatively little impact. More significant are climate‐induced variations in the isotopic discrimination by plants (e.g., Kaplan, Prentice, & Buchmann, 2002). Three natural sources of CH_4_ (wetlands, biomass burning, and termites) are potentially influenced by the isotopic signature of leaf carbon. For example, C3 and C4 plants exhibit very different leaf carbon isotope ratios. Termites are not observed to show any relationship between emitted *δ* ^13^CH_4_ and the proportion of C3 versus C4 plants (Tyler et al., 1988), so we do not further consider this source term.

Leaf *δ* ^13^C is a function of the isotopic discrimination at each stage of gas transfer from the ambient environment to the chloroplasts within the leaf where photosynthesis occurs (Lloyd & Farquhar, 1994). Changes in moisture stress and water use efficiency of a plant will influence the amount of time that stomata are open and hence the overall ratio of intercellular and ambient CO_2_. During the LGM, the lower CO_2_ significantly reduced plant water use efficiency and is thought to be responsible for around 15% of the total change in *δ* ^13^C between the two time periods (Kaplan, Prentice, Knorr, et al., 2002). The lower temperatures and hence potential evaporation and generally drier environment will have competing impacts on the plant available moisture (e.g., Scheff et al., 2017).

**Figure 2:**
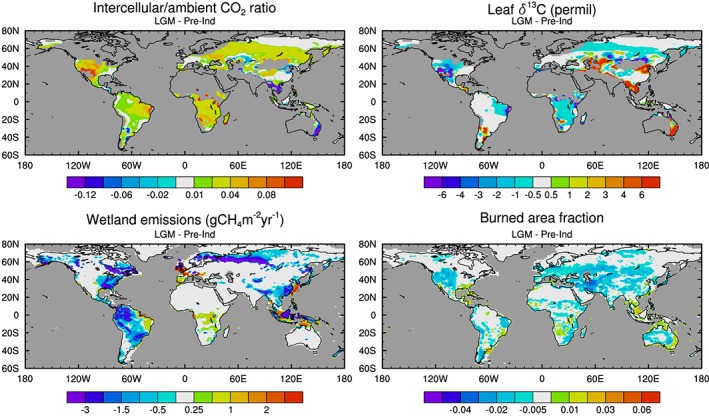
HadGEM2‐ES simulated Last Glacial Maximum minus preindustrial anomalies in intercellular/ambient CO_2_ ratio, leaf *δ* ^13^C (this study) and wetland CH_4_ emissions, and burned area fraction (from H17). The CO_2_ ratios and *δ* ^13^C fields are masked over desert and ice regions. Emissions and burned area anomalies are shown over new land points and where ice sheets change to clarify the role of geographical differences between the two time periods.

We performed late preindustrial and LGM atmosphere‐only simulations with HadGEM2‐ES similar to those reported previously (Hopcroft & Valdes, 2015; H17) but with new intercellular leaf CO_2_ diagnostics (implemented within the dynamic vegetation scheme of Cox (2001), see supporting information, to calculate the isotopic discrimination by vegetation for the two time periods (following Kaplan, Prentice, & Buchmann, 2002; Lloyd & Farquhar, 1994). The resultant leaf to ambient CO_2_ ratio and $\delta^{13}{\text{C}}_{\text{leaf}}$ anomalies are shown in Figure 2 (weighted by plant functional type fractional coverage). There is a global shift to less negative $\delta^{13}{\text{C}}_{\text{leaf}}$ values, from a global average of −26.3‰ to −25.9‰. This is dominated by positive changes in semiarid regions. A reduction in *δ* ^13^C during the LGM relative to the late preindustrial in South Africa is in agreement with the model‐data comparison of Bragg et al. (2013).

To decompose the drivers of the leaf *δ* ^13^C change, we used an updated version of the land surface component of HadGEM2‐ES (JULES v4.1: Harper et al., 2016), to quantify the relative influence from changes in climate, atmospheric CO_2_, and vegetation, shown in Figure S1. Compared to the globally averaged leaf *δ* ^13^C increase of 0.36‰ (which is close to the global average change simulated with HadGEM2), CO_2_, climate, and dynamic vegetation separately cause changes of −0.29‰, −0.11‰, and 0.21‰, respectively (and averaged over preindustrial land points only). The CO_2_ forced change dominates in the tropics, whereas climate is more significant in midlatitudes. Both of these factors lead to negative excursions for the LGM relative to the preindustrial. The vegetation distribution change has a more widespread and mixed influence, with regions of negative shifts (LGM relative to preindustrial) in leaf *δ* ^13^C in southern Africa, western north America, and Eurasia, and positive changes in South East Asia, South America, and Australia.

The isotopic signature of CH_4_ emissions was calculated from the weighted sum of the simulated monthly leaf *δ* ^13^C values, using either wetland CH_4_ emissions or monthly burned area (H17), see Figure 2. We retain the 4‰ difference in *δ* ^13^CH_4_ between tropical and extratropical wetlands and assumed no offset between *δ* ^13^ *C*_*leaf*_ and that of biomass burning (Chanton et al., 2000). We are unable to model this offset for wetland emissions, because, like most global models, JULES does not represent the production, transport, and oxidation of CH_4_ isotopes. The *δ* ^13^C shift is averaged regionally corresponding with the three‐box model.

The global mean wetland source increases by 0.7‰ and the biomass burning source increases by 1.6‰. For wetlands, the change in the distribution of emissions alone, mostly a relocation to the southern extratropics and tropics, causes a shift of 0.9‰. The leaf ^13^CH_4_ change alone induces a change of 3.3‰. The influence from the assumed offset between the tropical and extratropical wetlands was quantified by setting this difference to zero. It has a strong influence on the result: without this effect the overall signature change is negligible. We find that these separate changes do not combine linearly. For the biomass burning, the change in the global distribution of burning causes a 1.4‰ increase, while the leaf ^13^CH_4_ change alone causes a 1.3‰ increase.

The isotopic fractionation of CH_4_ shows a very small effect from the temperature dependence of fractionation during methanogenesis and methane consumption (following empirical evidence from Tyler et al., 1994) and as described in the supporting information (Blair et al., 1993; Conrad, 2005; Moosavi & Crill, 1998; Schaefer & Whiticar, 2008; Whiticar, 1999). The changes of −0.3‰ and −2‰ for the wetland signature and soil uptake fractionation factor respectively have a negligible impact on the *δ* ^13^CH_4_.

The calculated changes in the isotopic signature of wetland *δ* ^13^C averaged in three latitude bands (−0.1, 0.1, and 0.23‰ in the northern, tropical, and southern boxes, respectively) and similarly for biomass burning (2.6, −0.1, and 4.5‰) were included along with the small change in fractionation factors due to the temperature dependence of methanogenesis and uptake in the box model. The zonally averaged values can be summed to give the global total when weighted by changing sources strengths in the three bands, given in Table 1. The results are also shown in Figure 1. The simulated influence of leaf *δ* ^13^C on the atmospheric isotopic signature is relatively limited at 0.6‰. This does not capture a substantial fraction of the observed change and implies that changes to both the source mixture and the individual source signatures are required.

## 4 Inferring Source Changes From Ice Core Observations

To infer the CH_4_ sources that are consistent with the ice core observations, we employ a Bayesian framework (e.g., Denison et al., 2002). In this, information from ice cores is combined with the model simulations in a probabilistic formulation which accounts for the estimated uncertainties. We use the model simulations of H17 as prior information and condition the posterior probability density functions (PDFs) for the source strengths using the ice core observations. This approach differs from that of Fischer et al. (2008), because we incorporate prior information rather than sampling unconstrained.

We use a Metropolis‐Hastings Markov chain Monte Carlo algorithm (MCMC, see Gilks et al., 1995) to sample the posterior, conditioned on how well the model reproduces the LGM CH_4_ concentration and the preindustrial and LGM *δ* ^13^C and *δ*D(CH_4_) values. Assigned observational uncertainties are 2 ppbv, 0.3‰, and 4.0‰ for CH_4_, *δ* ^13^CH_4_, and *δ*DCH_4_, respectively (Mitchell et al., 2013; Möller et al., 2013; Sowers, 2006, 2010; WAIS Divide Project Members, 2015), see Table S1. These could be modified to account for representational uncertainty associated with the use of a coarse box model, but we have not done this here.

Uncertainty surrounding the isotopic signatures or fractionation factors for sources and sinks are taken into account by sampling from prior distributions. The mean values are detailed in supporting information Table S2 for sources (Etiope et al., 2008; Fisher et al., 2017; Miller, 2005; Sherwood et al., 2017; Snover et al., 2000; Thornton et al., 2016; Whiticar & Schaefer, 2007) and sinks (DeMore, 1993; Gierczak et al., 1997; Saueressig et al., 2001). The uncertainty estimates are given in Table S3 for sources (Etiope et al., 2008; Fischer et al., 2008; Quay et al., 1999; Sherwood et al., 2016; Snover et al., 2000) and sinks (Röckmann et al., 2011; Saueressig et al., 1995, 1996, 2001; Snover & Quay, 2000).

The prior information (summarized in Table 1) on the five methane source terms are normal distributions, with mean values equal to the LGM simulated values from H17, except for hydrate and geological sources for which the preindustrial values are used. The standard deviation of these prior distributions is set to the average of top‐down estimates of Kirschke et al. (2013). Assuming the quoted ranges are representative of the 95% limits, these equal 13%. We double this value to account for additional uncertainty for past time periods. Since modern biomass burning rates are influenced by anthropogenic activity (Bowman et al., 2009), we take a range from the literature of 14.3 TgCH_4_/year (Lamarque et al., 2010) and 37 TgCH_4_/year (Thonicke et al., 2005) and derive a normal distribution standard deviation of approximately 4 TgCH_4_/year. The geological and hydrates terms are also subject to wider uncertainties (Etiope et al., 2008; Petrenko et al., 2017. We assume that these terms have a similar relative uncertainty as biomass burning, that is, a standard deviation of 2.8 TgCH_4_/year.

Any change in biomass burning CH_4_ would be accompanied by a change in wildfire emission of nitrogen oxides and carbon monoxide which would impact on the CH_4_ lifetime. In H17 varying the LGM fire source between 10% and 84% of the late preindustrial source total, led to 2% and 8% relative increase in CH_4_ lifetime. Thus, we include this co‐variation.

**Figure 3:**
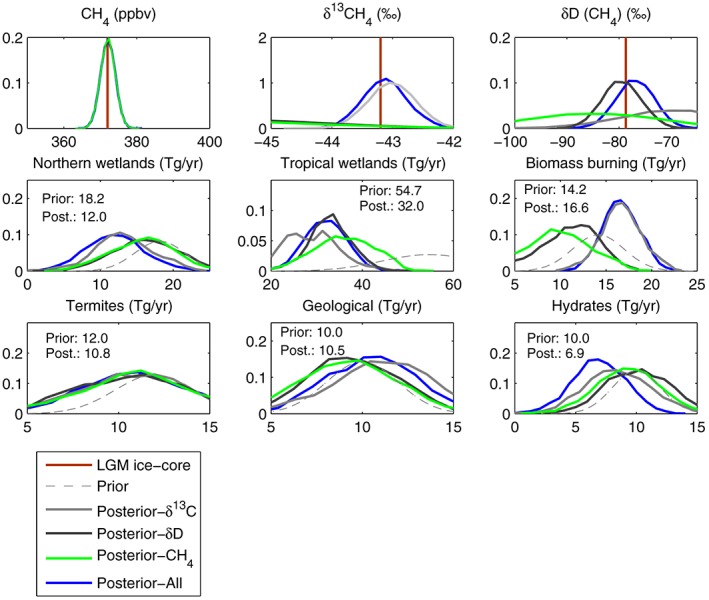
Posterior probability density functions of Last Glacial Maximum CH_4_ concentrations (ppbv), isotopes (‰), and emission rates (TgCH_4_/year) as inferred with the MCMC algorithm, with annotated prior and posterior mean values (for the all case as shown by blue curves). The four different posteriors show the impact of including all of the observations, the CH_4_ concentration alone or combined with the *δ* ^13^CH_4_ or the *δ*DCH_4_. The strongest overall constraint comes from the concentration itself, while the deuterium appears to have the weakest overall influence. The influence of *δ* ^13^CH_4_ mostly manifests as a subtle increase in the mean for both geological and biomass burning fluxes (compare light gray versus green curves). The posterior mean for the geological emissions is the only term for which the inferred Last Glacial Maximum value is close to or higher than the preindustrial value.

The MCMC algorithm was run for one million iterations, with the initial 250,000 iterations discarded (Gilks et al., 1995). The four cases in Figure 3 are using (i) only CH_4_ concentration observations (green), (ii) both the concentration and *δ* ^13^CH_4_ or (iii) *δ*DCH_4_, and (iv) the concentration and both isotopes (blue). The top three panels show the fit to the LGM ice core observations in each case. In the lower panels, the distributions of the model parameters are shown (i.e., inferred source strengths at the LGM). Prior distributions are shown by dashed lines.

The results show that the MCMC algorithm reproduces the observed LGM methane concentration, *δ* ^13^CH_4_ and *δ*D(CH_4_) well. All four cases point to a reduced tropical wetland CH_4_ flux at the LGM. The posterior mean derived by considering all observations is 32.0 ± 5.0 TgCH_4_/year (mean ±1 standard deviation), and this is 59% of the prior mean (54.7 ± 15 TgCH_4_/year), or 44% of the preindustrial value. Northern wetlands are inferred to decrease to 22% of the preindustrial value when all observations are included, a reduction of 33% relative to the prior distribution. The prior and posterior are less divergent for termite, geological, and hydrate source strengths. The geological term is the only source with an increase at the LGM (posterior mean is 10.5 ± 2.5 TgCH_4_/yr), prior (10.0 ± 2.8 TgCH_4_/year). This emerges in both the all data and the concentration with *δ* ^13^C cases. It is driven by the high *δ* ^13^CH_4_ of geological sources (−33‰) and the model‐informed prior distribution (H17) on biomass burning (the other *δ* ^13^CH_4_‐enriched source), which points to a reduction in biomass burning at the LGM.

In the remaining cases, the solutions that stem from only considering the CH_4_ concentration alone or with one isotope type, are caused by the interplay between satisfying the prior information and the observations. For example, in the CH_4_‐only case, both the biomass burning and geological terms are reduced, because the isotopic constraint is absent and because this allows the wetland source to stay nearer to its prior distribution.

The inferences drawn separately from the *δ* ^13^CH_4_ and *δ*D(CH_4_) are not entirely consistent. Including the concentration with deuterium leads to an increased hydrate flux and a reduced biomass burning term, while *δ* ^13^CH_4_ leads to the opposite behavior. Overall the reduced tropical wetlands are required to satisfy the concentration constraint, while relatively high biomass burning and geological sources are required by the *δ* ^13^CH_4_. The main constraint from *δ*D is more difficult to isolate. It acts in a compensatory manner between the biomass burning and hydrate terms.

Doubling the observational or prior uncertainty estimates has very little impact on the posterior distributions (Figures S2 and S3). With uniform priors (Figure S4), the posterior distributions show even stronger reductions in both extratropical and tropical wetlands (for example, the posterior mean for tropical wetlands is 61% lower than the prior mean). The termite term is significantly higher than assumed with the original prior, but this is inconsistent with the best estimate for the LGM emission strength (H17). This demonstrates the potential value of introducing prior information. We also tested the approach by using the prior for the LGM but with the CH_4_ concentration and isotope observations replaced with the late preindustrial values (see Figure S5). Overall, the sensitivity tests compared in Figures S2–S5, support the main conclusions above.

## 5 Discussion

Using recent process‐based Earth Model simulations of the late preindustrial and LGM methane budget, we calculate the likely impact on atmospheric CH_4_ isotopes. This shows that current estimates of the methane cycle underestimate both the concentration change and the isotopic response, even accounting for uncertainty in LGM biomass burning emissions.

We extend previous work (Kaplan, 2002; Ringeval et al., 2013; H17) to calculate the potential influence of leaf *δ* ^13^C of terrestrial vegetation on the natural sources of CH_4_ (i.e., wetlands and biomass burning). Though the results are likely model dependent (because they are sensitive to the spatial distribution of emissions), they suggest a limited role for changes in the isotopic discrimination of vegetation in the glacial‐interglacial changes in *δ* ^13^CH_4_. This raises the question of why there is such a clear correlation between *δ* ^13^CH_4_ and CO_2_ in the ice core record, which Möller et al. (2013) have attributed in part to the influence of leaf ^13^C on the emissions of CH_4_ from tropical wetlands.

We also found that climatically driven changes in the boundary layer Cl sink contributed only 0.12‰ to the *δ* ^13^CH_4_ at the LGM relative to the preindustrial, consistent with past work (Levine, Wolff, Jones, & Sime, 2011). A substantial increase in sea salt aerosols at high latitudes during the LGM (Fischer et al., 2007) may have enhanced the Cl sink (Levine, Wolff, Jones, & Sime, 2011), but this is yet to be included in models (Levine et al., 2014). We have also not included any terrestrial sources of CH_3_Cl.

We used a probabilistic approach to show that both biomass burning and geologic sources of methane may have played a role in setting the observed enrichment of atmospheric CH_4_ (Bock et al., 2017). The latter is supported by the proposed sea level control on marine seepage of CH_4_ (Etiope et al., 2008; Luyendyk et al., 2005). Hence, our modeling results are potentially consistent with the Möller et al. (2013) observations because of the high degree of covariance between CO_2_, sea level, and other climatically relevant variables on a glacial‐interglacial timescale.

Further, the estimated modern day natural geological CH_4_ source of around 54 TgCH_4_/year (Kirschke et al., 2013) cannot be reconciled with the required reduction in total methane sources at the LGM (H17). This is because the geological source likely increased at the LGM and because 54 TgCH_4_/year is a substantial fraction of the natural source. Recent mass balance calculations based on late glacial ^14^CH_4_ measurements (Petrenko et al., 2017) also support this.

The inferred large reduction in wetland emissions is not simulated by H17 and is at the extreme limit of the uncertainty range of recent model‐based estimates of the LGM‐PI wetland change, (29% to 67%: Hopcroft et al., 2014; Ringeval et al., 2013; Weber et al., 2010). However, several processes (e.g., wetland carbon cycle processes, nutrient status, and tree‐mediated transport) that are not properly represented (Melton et al., 2013; Pangala et al., 2017; Wania et al., 2013; H17) need to be evaluated.

## 6 Conclusions

Current process‐based estimates suggest that a 50% reduction in sources is required to explain the CH_4_ concentration during the LGM. Bottom‐up estimates of emissions fail to replicate this. A comprehensive Earth System model study (H17) also underestimates the changes in CH_4_ stable isotopes. Accounting for changes in isotopic discrimination of vegetation does not explain the observed LGM‐PI *δ* ^13^CH_4_ shift.

We applied a Bayesian framework to resolve the potentially conflicting information from models and observations. The results suggest that the concentration change was predominantly driven by wetlands, but the inferred emissions response is at the extreme end of model predictions. The isotopic change is either driven by a relatively limited reduction in biomass burning at the LGM, perhaps brought about by human activities (Kaplan et al., 2016), or an increase in nonhydrate geological CH_4_ emissions. Future work needs to better understand the climatic sensitivity of natural CH_4_ sources, including wetland and geological terms, and this study suggests that incorporation of CH_4_ stable isotopes into Earth System model simulations is one avenue for further progress.

## Supporting information



Supporting Information S1
