# Supplementary material for: Bayesian Analysis of the Glacial‐Interglacial Methane Increase Constrained by Stable Isotopes and Earth System Modeling
Source: Geophys Res Lett. 2018 Apr 22;45(8):3653–63. doi: 10.1002/2018GL077382 (PMC6001704; doi:10.1002/2018GL077382)
Supplement: Supplementary file 1 — Supporting Information S1 [file GRL-45-3653-s001.pdf]

Supporting Information for "Bayesian analysis of the glacial-interglacial methane increase constrained by stable isotopes and Earth System modelling"

Peter O. Hopcroft[1,2,3], Paul J. Valdes[1,2] & Jed O. Kaplan[4,5]

Corresponding author: P. O. Hopcroft, School of Geography, Earth and Environmental Science, University of Birmingham, Edgbaston, U.K. (p.hopcroft@bham.ac.uk)

[1]Bristol Research Initiative for the

1. HadGEM2-ES simulations

HadGEM2-ES is a coupled general circulation model [*HadGEM2 Development Team*, 2011] with interactive Earth System components that has been widely used to study past [*Kandlbauer et al.*, 2013; *Hopcroft and Valdes*, 2015], present [*Booth et al.*, 2012] and future climate change [*Caesar et al.*, 2013], including as part of CMIP5 [*Jones et al.*, 2011]. HadGEM2-ES incorporates schemes for vegetation [*Cox*, 2001], wetlands [*Gedney et al.*, 2004], tropospheric aerosols and tropospheric chemistry [*O'Connor et al.*, 2014]. The full ES configuration [*Collins et al.*, 2011] includes coupled 3D atmosphere and ocean

Dynamic Global Environment, School of Geographical Sciences, University of Bristol, U.K.

[2]Cabot Institute, University of Bristol, U.K.

[3]Now at the School of Geography, Earth and Environmental Sciences, University of Birmingham, Edgbaston, U.K.

[4]Max Planck Institute for the Science of Human History, Jena, Germany.

[5]ARVE Research SARL, Pully, Switzerland.

general circulation models and a dynamic sea-ice scheme [*HadGEM2 Development Team*, 2011]. Here we utilise an atmosphere-only version in which sea surface temperature (SST) fields and sea-ice are prescribed based on pre-industrial and last glacial maximum (LGM) simulations with HadCM3 [*Singarayer and Valdes*, 2010].

Leaf internal CO_2 is not routinely output by the model, but is used in the calculation of stomatal conductance and photosynthesis [*Cox*, 2001]. We used this parameter in the formulation of *Lloyd and Taylor* [1994] to predict the isotopic carbon discrimination of terrestrial vegetation within the model for the pre-industrial and LGM time periods. For this we followed the implementation of *Kaplan et al.* [2002]. These simulations are otherwise identical to the atmosphere-only (without chemistry simulations) reported by *Hopcroft et al.* [2017].

2. Offline CH_4 sources used in HadGEM2-ES

Wetland CH_4 emissions are computed within HadGEM2-ES at each model timestep [*Collins et al.*, 2011]. Wetland area is calculated with a TOPMODEL scheme [*Gedney et al.*, 2004] and configured with a recently derived high-resolution topographic index dataset [*Marthews et al.*, 2015], which quantifies the propensity of the topography for subgrid areas of soil moisture saturation.

Emissions from biomass burning were simulated using LPJ-LMfire [*Pfeiffer et al.*, 2013] and were scaled to give a pre-industrial flux of $21 \mathrm{TgCH_4 yr^{-1}}$ This represents a 50% increase on the value used by H17 which was taken from the pre-industrial value for 1850 used in CMIP5. This higher value is consistent with $25 \mathrm{TgCH_4 yr^{-1}}$ inferred by *Ferretti et al.* [2005] but is lower than some model-based estimates [e.g. *Thonicke et al.*, 2005,

simulated a pre-industrial flux of 37 $TgCH_4yr^{-1}$]. This higher value also gives a superior fit to observed CH_4 isotopic data for the pre-industrial simulation, as compared with using the value of 14.3 $TgCH_4yr^{-1}$ from H17.

We considered three scenarios of LGM biomass burning fluxes. The first is termed standard and follows the simulations using LPJ-LMfire for the pre-industrial and LGM. The second is low-fire and sets all fire emissions at the LGM to 10% of the pre-industrial fluxes. The final employs a different version of LPJ-LMfire in which an estimated human contribution to biomass burning during the LGM is included following the methods described by *Kaplan et al.* [2016].

The ocean CH_4 emissions used by *Hopcroft et al.* [2017] were scaled down to $1TgCH_4yr^{-1}$ in line with more recent estimates [*Kirschke et al.*, 2013]. An empirically-based method to predict termite CH_4 emissions as a function of vegetation cover based on data of *Sanderson* [1996] was employed using simulations with the BIOME4 vegetation model [*Kaplan et al.*, 2003]. The pre-industrial source was scaled to $20TgCH_4yr^{-1}$. The hydrate CH_4 source was set to $10TgCH_4yr^{-1}$ following previous studies [*O'Connor et al.*, 2014]. We include a further non-hydrate geological term to account for sources identified by *Etiope et al.* [2008], and set this to equal the hydrate flux.

To address uncertainty in the representation of wetland processes, we also ran offline simulations using a peatland and permafrost model for northern hemisphere extra-tropical CH_4 emissions [*Wania et al.*, 2010]. This gives a stronger pre-industrial extra-tropical methane flux, but also leads to a larger relative change at the LGM.

3. Atmospheric CH_4 box model

We use a three box model of tropospheric CH_4 concentration and its two isotopes similar to the two box model presented by *Miller* [2005]. Three equal area boxes have boundaries at approximately 20°S and 20°N. These boundaries are used to calculate regional averages from HadGEM2-ES coupled model simulations that are fed into the box model. The applied box model formulation yields six unknowns and six equations:

$$\frac{dB_N}{dt} = S_N - k_{12}B_N - k_{ex}(B_N - B_T) \quad (1)$$
$$\frac{dB_T}{dt} = S_T - k_{12}B_T - k_{ex}(B_T - B_N) - k_{ex}(B_T - B_S) \quad (2)$$
$$\frac{dB_S}{dt} = S_S - k_{12}B_S - k_{ex}(B_S - B_T) \quad (3)$$
$$(4)$$

and

$$\frac{d\delta_N}{dt} = \frac{\Sigma_N^i}{B_N} - \epsilon k_{12} - \frac{S_N\delta_N}{B_N} + k_{ex}\frac{B_T}{B_N}(\delta_N - \delta_T) \quad (5)$$
$$\frac{d\delta_T}{dt} = \frac{\Sigma_T^i}{B_T} - \epsilon k_{12} - \frac{S_T\delta_T}{B_T} + k_{ex}\frac{B_N}{B_T}(\delta_T - \delta_N) + k_{ex}\frac{B_S}{B_T}(\delta_T - \delta_S) \quad (6)$$
$$\frac{d\delta_S}{dt} = \frac{\Sigma_S^i}{B_S} - \epsilon k_{12} - \frac{S_S\delta_S}{B_S} + k_{ex}\frac{B_T}{B_S}(\delta_S - \delta_T) \quad (7)$$

Here B is atmospheric burden ($TgCH_4$), S is the source term ($TgCH_4yr^{-1}$), k_{12} is the inverse of atmospheric lifetime (yr^{-1}), k_{ex} is the exchange timescale between the boxes (=1.0 yr^{-1}). ϵ is the atmospheric fractionation term, δ is the CH_4 isotopic signature (‰) and Σ_N^i is the source weighted isotopic signature of all of the source terms (‰). The

subscripts N, T and S refer to the northern, tropical and southern boxes. Steady state is assumed in each time period, so the left hand terms are set to zero.

This treatment introduces a small approximation from not treating the two isotopes separately [*Lassey et al.*, 2000] that is likely small compared with other uncertainties.

The global mean surface CH_4 concentration is calculated as the sum of the burden (Tg) in each box divided by k (=2.75 $TgCH_4ppbv^{-1}$). k reduced by 2.7% for the LGM following *Hopcroft et al.* [2017]. For comparison with the observations, the northern or southern box are compared with the ice-core data from Greenland or Antarctica respectively, as listed in table S1.

4. Potential effect of temperature-dependent fractionation during methanogenesis and soil uptake

The isotopic composition of wetland CH_4 is a function of leaf $\delta^{13}C$ (as the substrate for methanogensis), the methanogenesis pathway and other environmental factors [*Whiticar*, 1999]. The dependence of fractionation is not well understood [*Conrad*, 2005; *Schaefer and Whiticar*, 2008]. There is evidence of a temperature dependence of isotopic fractionation in the production of CH_4 via the carbonate reduction pathway [*Blair et al.*, 1993], but the isotopic kinetic effect is not well understood for methyl fermentation pathways, and the balance between these two pathways and the response of this balance to global climate change remains highly uncertain. Additionally, field experiments suggest that the ratio of net to gross production of CH_4 is insensitive to temperature [*Moosavi and Crill*, 1998], and hence we do not consider this as a factor that modifies the CH_4 isotopic signature for the glacial to interglacial change.

The fractionation of the carbonate reduction component of global wetland methanogenesis is temperature dependent. We used equation 2 of *Schaefer and Whiticar* [2008] with monthly simulated surface air temperatures and wetland CH_4 emissions in HadGEM2 late pre-industrial and LGM simulations. Assuming a carbonate reduction fraction of 30% of total emissions in both time periods, we obtain a change in wetland isotopic signature of -0.3 ‰. This is at the lower end of the values calculated using estimated global mean temperature changes by *Schaefer and Whiticar* [2008]. This has a very small impact on the global $\delta^{13}CH_4$ result.

We use the monthly simulated surface air temperatures in HadGEM2 and monthly soil uptake rates (from H17) for the late pre-industrial and LGM to calculate the effect of temperature on fractionation during soil uptake. This uses the empirically-derived relationship from fig 2a of *Tyler et al.* [1994]. This gives late pre-industrial and LGM soil uptake KIEs of -1.0190 and -1.0210, compared with estimates modern range of [-1.017, -1.022] and a LGM value of -1.0272 [*Whiticar and Schaefer*, 2007]. Because of the small contribution of the soil sink to the overall budget, this fractionation change of -2 ‰ has a negligible impact on the atmospheric $\delta^{13}CH_4$.

5. JULES simulations of carbon isotope discrimination by global vegetation

We used the Joint UK Land Environment Simulator (JULES) version 4.1 which has 9 plant functional types [*Harper et al.*, 2016]. JULES was driven with 3-hourly climate forcing for surface air temperature, precipitation, air pressure, wind speed, specific humidity and diffuse fraction of incident radiation, as archived from pre-industrial and LGM simulations with HadGEM2-ES. Atmospheric CO_2 was prescribed as in the HadGEM2-ES

simulations for the pre-industrial (280 p.p.m.) or LGM (185 p.p.m.). After an 80 year spin up, the dynamic vegetation is deactivated so that the distribution of vegetation types does not evolve in response to the sensitivity simulations.

An ensemble of 8 simulations was performed:

1. Pre-industrial
2. LGM
3. Pre-industrial with LGM vegetation distribution
4. Pre-industrial with LGM CO_2
5. Pre-industrial with LGM climate
6. LGM with pre-industrial CO_2
7. LGM with pre-industrial climate
8. LGM with pre-industrial vegetation distribution

For LGM with pre-industrial vegetation, the new land areas at the LGM (i.e. exposed continental shelves) are prescribed as a combination of 25% broadleaf trees, grasses, shrubs and bare soil. For the pre-industrial with LGM vegetation, the LGM ice-sheet areas are prescribed as bare soil.

The influence of a given variable x (= climate, CO_2, etc) is quantified relative to either base state (PI or LGM) as:

$$\Delta\delta^{13}C(\mathrm{x})_{PI} = \Delta\delta^{13}C(\mathrm{PI\ with\ LGM\ x}) - \Delta\delta^{13}C(\mathrm{PI}), \quad (8)$$

$$\Delta\delta^{13}C(\mathrm{x})_{LGM} = \Delta\delta^{13}C(\mathrm{LGM}) - \Delta\delta^{13}C(\mathrm{LGM\ with\ PI\ x}) \quad (9)$$

The results of these calculations are shown in figure S1 and are discussed in the main text.

6. Sensitivity tests with the Bayesian algorithm

6.1. Observational uncertainties

The observational uncertainties listed in table S1 do not account for uncertainties in translating modelled values to the real world point locations. We therefore re-ran the Bayesian algorithm with these uncertainties values doubled (figure S2). The resulting PDFs are compared with the default values presented in the main text in figure 3. In this case the assumed two sigma is 24% and 85% of the LGM to late pre-industrial difference for $\delta^{13}CH_4$ and deuterium, respectively.

With these larger uncertainty estimates, the model is much less able to reproduce the observed $\delta^{13}CH_4$ and deuterium values, and as a result infers a larger biomass burning reduction and little change in the geological flux.

6.2. Prior uncertainties

The impact of the choice of prior uncertainty terms was tested by doubling these values for each source (figure S3). This results in much wider posterior distributions. The strong reduction in wetland terms is consistent, but now the biomass burning flux increases slightly, with a posterior mean of 17.0±3.4 $TgCH_4yr^{-1}$, whilst the posterior standard deviations of the geological and termite terms increase by 70-80%. However, with a posterior mean of 10.5 $TgCH_4year^{-1}$, the inferred geological term is identical to the default example which is close to the pre-industrial flux, whilst the inferred mean biomass burning flux is still only 81% of the pre-industrial value.

With uniform priors, the posterior distributions show even stronger reductions in both extra-tropical and tropical wetlands (for example, the posterior mean for tropical wetlands is 61% lower than the prior mean). The posterior distributions for biomass burning and geological emissions are qualitatively different with the use of uniform prior distributions. However, with much wider posterior distributions (standard deviations increase by 1.8 $Tgyr^{-1}$ for hydrates and 3.8 $Tgyr^{-1}$ for termites), the mean values are rather similar to those presented in figure 3. The termite and hydrate terms are inferred to be significantly higher and lower respectively, than assumed with the original Gaussian prior, but the termite result is inconsistent with the best estimate for the LGM emission strength (H17) in which termite emissions are modelled to reduce substantially at the LGM because of reduced tropical forest coverage.

6.3. Testing with pre-industrial observations

The Bayesian Markov chain Monte Carlo (MCMC) methodology was also tested by imposing the LGM priors but using pre-industrial CH_4 concentration and isotope observations instead of those for the LGM. That is, the algorithm is given LGM prior information but is conditioned on pre-industrial observations.

This prior constitutes LGM values for wetlands, biomass burning and termites, and pre-industrial values for the remaining sources (hydrates and geological). The modelled LGM shift in isotopic signatures of wetland and biomass burning CH_4 were omitted.

The results are shown in figure S5. In this case the Bayesian inference recovers the observations. Interestingly the algorithm does not fully reconstruct the pre-industrial biomass burning source, and instead infers too strong a wetland source for the tropics.

This could be caused by imposing the LGM prior information. It illustrates the limits of this inference process because of the limited number of observations that are constraining the algorithm.

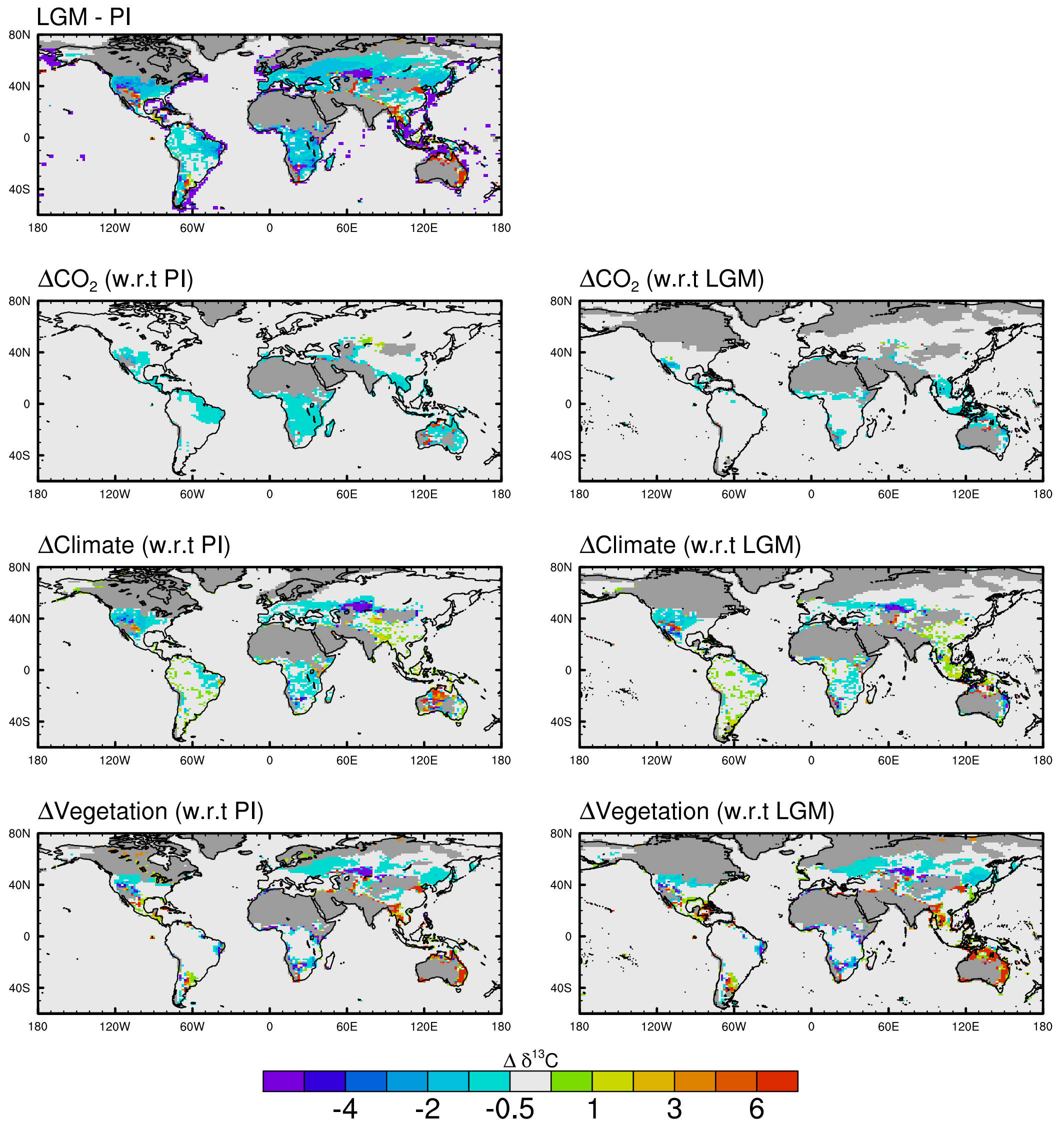

Figure S1. Offline JULES simulations of leaf $\delta^{13}C$ and the separate effects of climate, CO_2 and the vegetation distribution. The calculations are performed relative to the pre-industrial state (left column) or the LGM state (right column). Gridcells dominated by glaciers and deserts are masked.

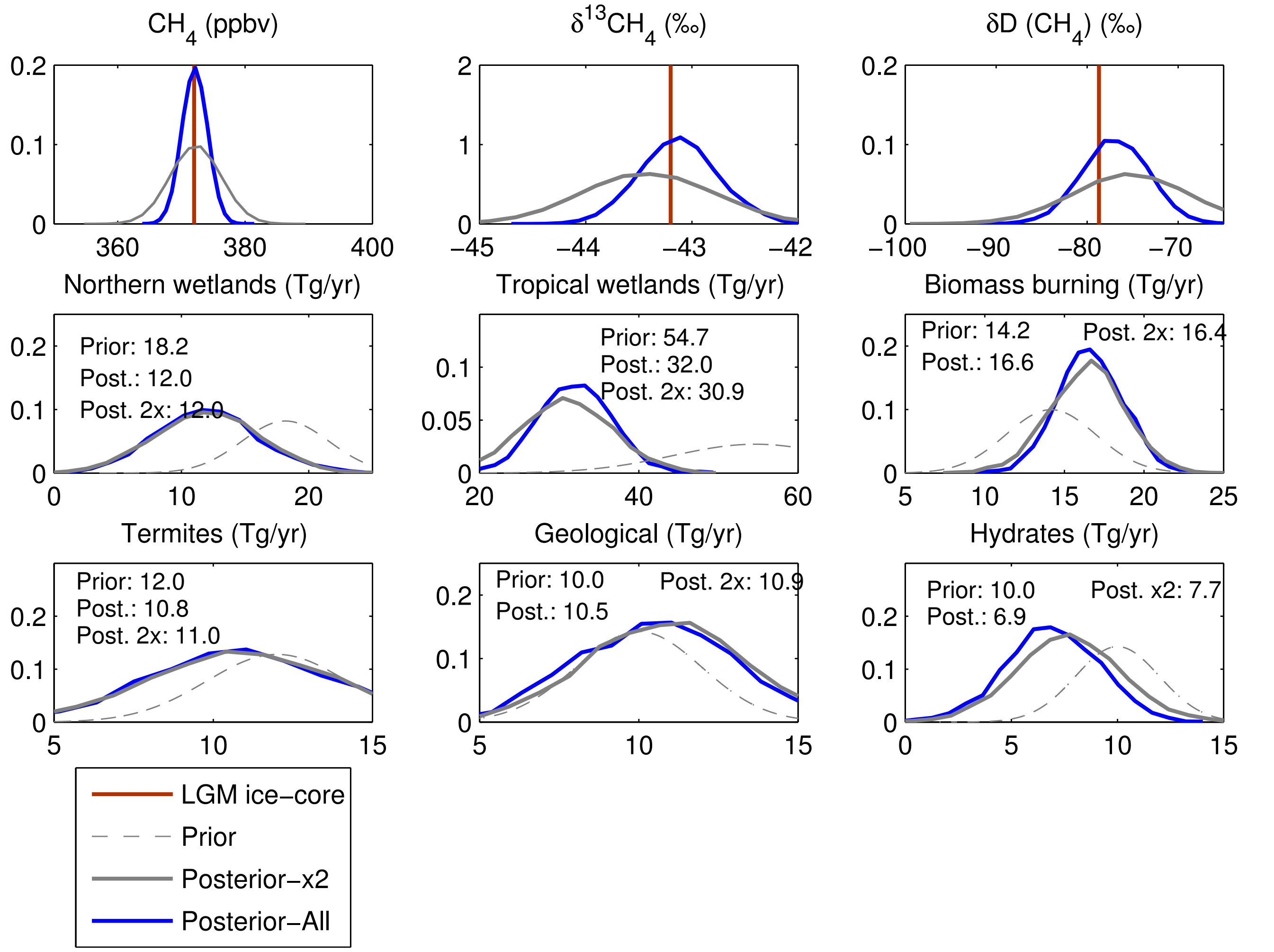

Figure S2. As in figure 3 of the main text, but comparing with a case where the observational uncertainties are doubled for both the late pre-industrial and the LGM (labelled as -x2). The default results reproduced from figure 3 of the main text are shown in blue for comparison.

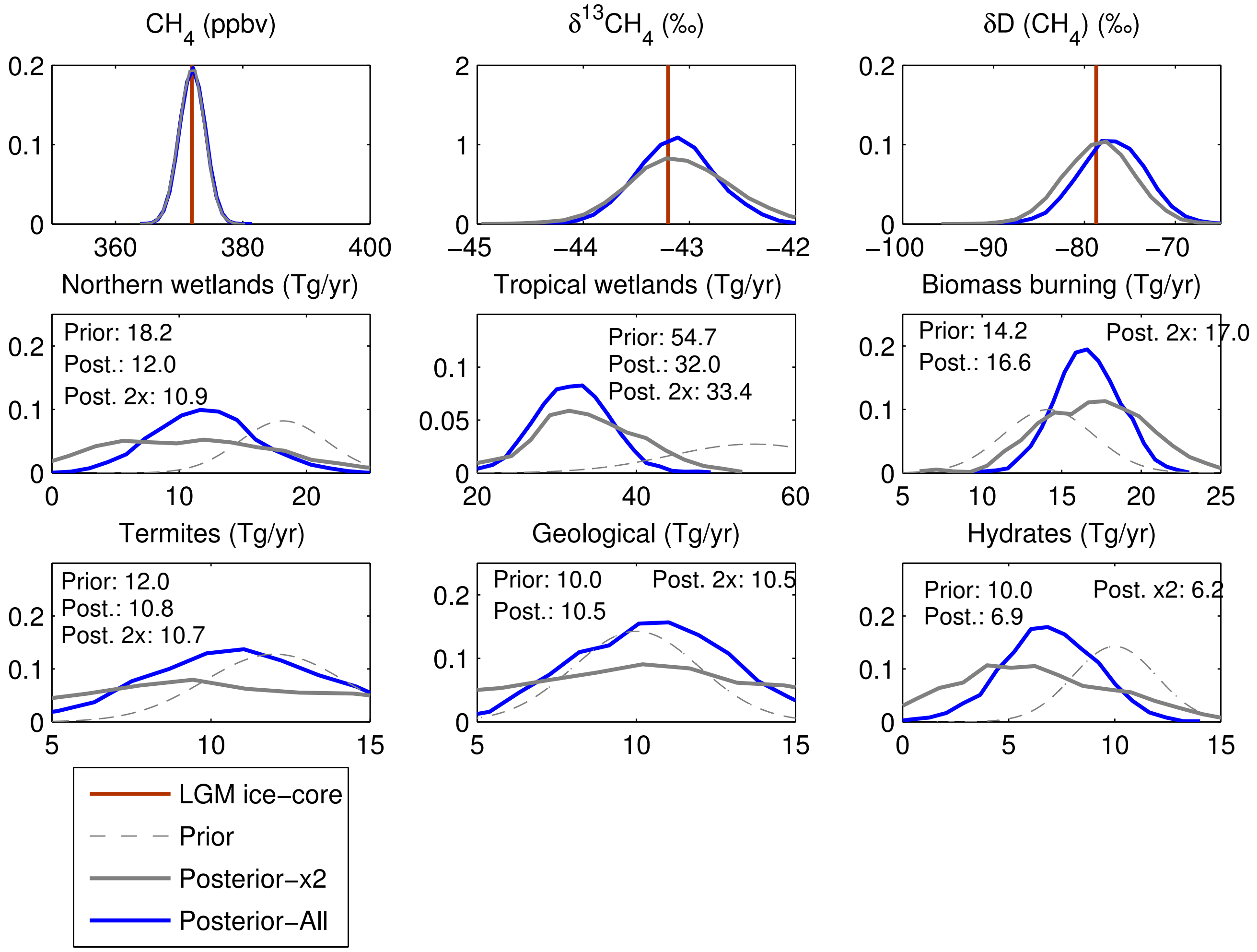

Figure S3. As in figure 3 of the main text, but comparing with a case where the uncertainties in the prior distributions on the CH_4 source terms are doubled (labelled as -x2). The default results reproduced from figure 3 of the main text are shown in blue for comparison.

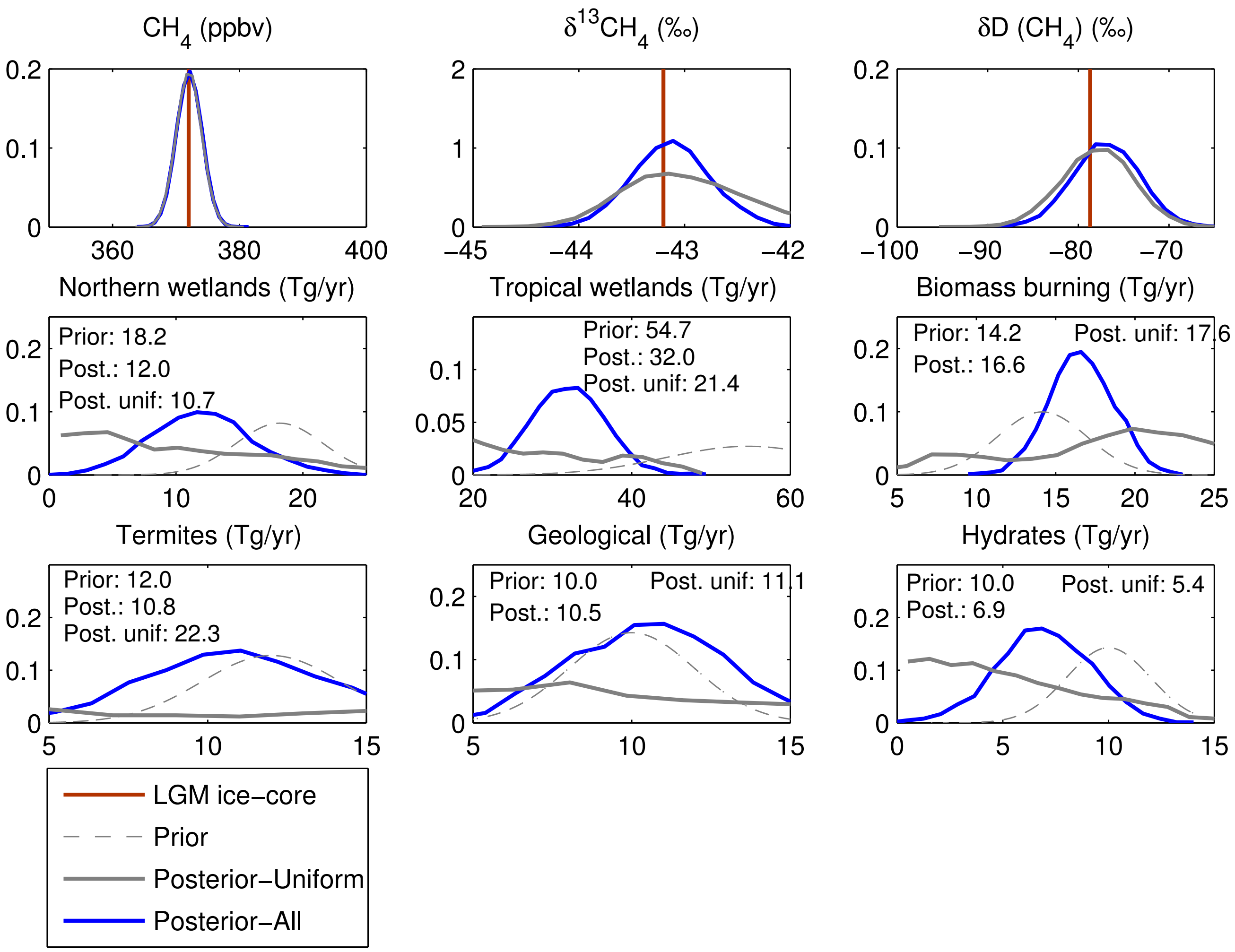

Figure S4. As in figure 3 of the main text, but comparing the default case with that using uniform prior distributions for all source terms with ranges (0,100) Tg/year for wetlands and (0,40) Tg/yr for other sources. The default results reproduced from figure 3 of the main text are shown in blue for comparison.

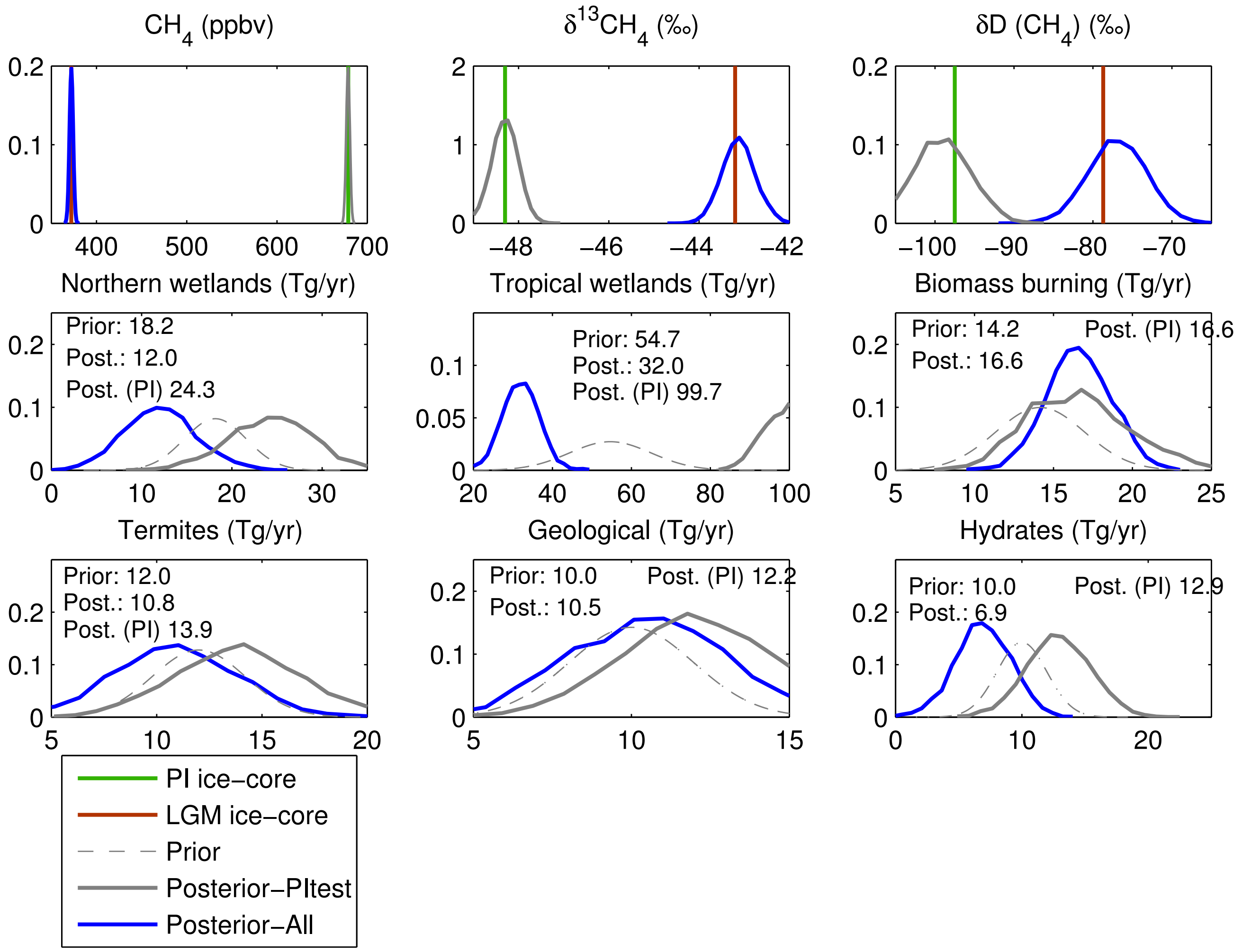

Figure S5. As in figure 3 of the main text, but now using pre-industrial observations and LGM prior information described in the main text (labelled as -PItest). The default results reproduced from figure 3 of the main text are shown in blue for comparison.

Table S1. Paleo- methane measurements from ice-cores, with timings, core name and the location in the three box model (North or South) and estimated measurement uncertainties.

Time	Age (kyr BP)	Mean	±1 s.d.	Ice core (Box)	Reference
**CH_4 (ppbv)**					
PI	0.4-1.2	679	2	WAIS (S)	*Mitchell et al.* [2013]
LGM	19.0-23.0	372	2	EDC (S)	*WAIS Divide Project Members* [2015]
**$\delta^{13}CH_4$(‰)**					
PI	0.4-1.2	-48.3	0.3	GISP2 (N)	*Sowers* [2010]
LGM	19.3-22.4	-43.2	0.3	EDML (S)	*Möller et al.* [2013]
**$\delta D(CH_4)$(‰)**					
PI	0.4-1.2	-97.5	3.0	GISP2 (N)	*Sowers* [2010]
LGM	19.1-23.1	-78.7	4.2	GISP2 (N)	*Sowers* [2006]

Table S2. Methane source and sink isotopic signature mean values and references.

	$\delta^{13}CH_4$	References	$\delta D(CH_4)$	References
Extra-trop. wetland	-62	*Thornton et al.* [2016]; *Sherwood et al.* [2017]<br>*Fisher et al.* [2017]	-375[a]	*Whiticar and Schaefer* [2007]; *Sherwood et al.* [2017]
Tropical wetland	-58	*Sherwood et al.* [2017]	-360[a]	*Whiticar and Schaefer* [2007]; *Sherwood et al.* [2017]
Biomass burning	-23	*Snover et al.* [2000]	-211	*Sherwood et al.* [2017]
Biomass burning		*Sherwood et al.* [2017]		
Termites	-57	*Miller* [2005]	-343	*Sherwood et al.* [2017]
Oceans	-58	*Miller* [2005]	-220	*Whiticar and Schaefer* [2007]
Hydrates	-62.5	*Whiticar and Schaefer* [2007]	-190	*Whiticar and Schaefer* [2007]
Other geological*	-33	*Etiope et al.* [2008]	-200	*Whiticar and Schaefer* [2007]
OH oxidation	-3.9	*Saueressig et al.* [2001]; *Gierczak et al.* [1997]; *Saueressig et al.* [2001]	-233	*DeMore* [1993]
Soil uptake	-18	*Snover and Quay* [2000]	-80	*Snover and Quay* [2000]
Stratospheric loss	-15.5	*Röckmann et al.* [2011]	-152	*Röckmann et al.* [2011]
Cl	-66.0	*Saueressig et al.* [1995]	-508	*Saueressig et al.* [1996]

[a] Tropical value following *Whiticar and Schaefer* [2007], and the offset of -15‰ for extra-tropical wetlands follows the regional averages in the data compilation of *Sherwood et al.* [2017].
* calculated using mean values as a weighted sum of estimated source strength and measured isotope values for mud volcanoes, microseepage, geothermal and marine seeps.

Table S3. Methane source and sink isotopic signature uncertainty estimates (±1 s.d.) and references.

	$\delta^{13}CH_4$	References	$\delta D(CH_4)$	References
Extra-trop. wetland	±5	*Sherwood et al.* [2016]	±20	*Quay et al.* [1999]
Tropical wetland	±5	*Sherwood et al.* [2016]	±20	*Quay et al.* [1999]
Biomass burning	±3	*Sherwood et al.* [2016]	±16	*Snover et al.* [2000]
Termites	±3	*Sherwood et al.* [2016]	±20	As wetlands
Oceans	±5	As wetlands	±20	As wetlands
Hydrates	±7	*Fischer et al.* [2008]	±5	*Fischer et al.* [2008]
Other geological*	±5	*Etiope et al.* [2008]	±5	as hydrates
OH oxidation	±0.2	*Saueressig et al.* [2001]	±9	*Saueressig et al.* [2001]
Soil uptake	±1	*Snover and Quay* [2000]	±30	*Snover and Quay* [2000]
Stratospheric loss	±1.2	*Röckmann et al.* [2011]	±6	*Röckmann et al.* [2011]
Cl	±2	*Saueressig et al.* [1995]	±31	*Saueressig et al.* [1996]

* calculated using ranges with a weighted sum of estimated source strength and measured isotope values for mud volcanoes, microseepage, geothermal and marine seeps.

Table S4. Methane sources and sinks and the prescribed isotopic signatures and the weighted source and sink isotopic signatures.

	Late Pre-Ind	LGM	ΔLGM
Sources ($TgCH_4yr-1$)		mean	%
N. extra-tropical wetland	55	18	-67
Tropical wetland	73	55	-25
S. extra-tropical wetland	11	13	18
Biomass burning	21	13.5(2,18)	-36(-90,-14)
Termites	20	12	-40
Oceans	1	0.8	-20
Hydrates	10	10	0
Other geological*	10	10	0
Sum	201	139	-31
Source weighted δ^{13}C(‰)	-53.3	-51.2	-
Source weighted D(‰)	-322.7	-307.4	-
Sinks (yr)			
OH oxidation	10.4	11.2[a]	6
Soil uptake	480	536	+12
Stratospheric loss	298	286	-4
Marine boundary layer Cl	961	834	-13
Total lifetime	9.7	10.3[b]	5
Weighted δ^{13}C fractionation (‰)	-5.2	-5.4	-
Weighted δD fractionation (‰)	-230.0	-230.5	-

Refer to tables S2 and S3 for references.
[a] The change in fire is used to determine the lifetime increase at the LGM following the three simulations for which fire CH_4 emissions for the LGM were prescribed as 10%, 66% and 84% of the late pre-industrial flux, leading to OH lifetime changes (LGM-PI) of +2.2, +5.6 and +7.8% respectively.
[b] Shows standard-fire scenario value, lowfire=9.9 yr, standard+LGM humans=10.4 yr.
